# Challenges in the care of patients with Chagas disease in the Brazilian public health system: A qualitative study with primary health care doctors

**DOI:** 10.1371/journal.pntd.0008782

**Published:** 2020-11-09

**Authors:** Renata Fiúza Damasceno, Ester Cerdeira Sabino, Ariela Mota Ferreira, Antonio Luiz Pinho Ribeiro, Hugo Fonseca Moreira, Thalita Emily Cezário Prates, Cristina Andrade Sampaio, Desirée Sant´Ana Haikal

**Affiliations:** 1 Program in Health Sciences, State University of Montes Claros (Universidade Estadual de Montes Claros), Montes Claros, Minas Gerais, Brazil; 2 Institute of Tropical Medicine, University of São Paulo (Universidade de São Paulo), São Paulo, São Paulo, Brazil; 3 Department of Internal Medicine, Federal University of Minas Gerais (Universidade Federal de Minas Gerais), Belo Horizonte, Minas Gerais, Brazil; 4 Point Data Research and Consulting, Montes Claros, Minas Gerais, Brazil; UNITED STATES

## Abstract

**Background:**

Care to patients with Chagas disease (CD) is still a challenge for health systems in endemic and non-endemic countries. In the Brazilian public health system, the expansion of Primary Health Care (PHC) services to remote and disadvantaged areas has facilitated the access of patients with CD to medical care, however this is in a context where care gaps remain, with insufficient public funding and inadequate distribution of services. Considering the need for studies on care to patients with CD in different settings, this study explored the challenges of family doctors to provide care to patients with CD in an endemic region in Brazil with high coverage of public PHC services.

**Methods and findings:**

This is a qualitative study. A focus group with 15 family doctors was conducted in a municipality participating in a multicenter cohort that monitors almost two thousand patients with CD in an endemic region in Brazil. The data were analyzed using a thematic content analysis technique. The family doctors pointed out the following challenges for care to patients with CD: unsatisfactory medical training (academic education not suitable for the clinical management of the disease, and lack of training on CD in PHC); uncertainties regarding antiparasitic treatment in the chronic phase of the disease; difficulty in patients’ access to specialized care when necessary, especially to the cardiologist; and trivialization of the disease by patients as a barrier to seeking care.

**Conclusion:**

The access of CD patients to adequate medical care, even in regions with high coverage of public PHC services, still represents an important challenge for health systems. The results of this study may contribute to the development of strategies to improve the clinical management of CD in PHC.

## Author summary

Chagas disease (CD) is a parasitic disease caused by *Trypanosoma cruzi* which predominantly affects poor and vulnerable populations. In the Brazilian public health system, the expansion of Primary Health Care (PHC) services to remote and disadvantaged areas has facilitated the access of patients with CD to medical care. However, preliminary results from a cohort study that followed almost two thousand CD patients in an endemic region in Brazil showed that 74% of these patients were not followed by a doctor or were followed irregularly. Our study explored the challenges of family doctors to provide care to patients with CD in a municipality in that region through a qualitative approach. We conducted a focus group with 15 family doctors. The family doctors pointed out the following challenges for care to CD patients in PHC: unsatisfactory medical training; uncertainties regarding antiparasitic treatment in the chronic phase of the disease; difficulty in patient access to specialized care when necessary; and trivialization of the disease by patients as a barrier to seeking care. The results of this study may contribute to the development of strategies to improve the clinical management of CD in PHC.

## Introduction

Chagas disease (CD) is a parasitic disease caused by *Trypanosoma cruzi* (*T*. *cruzi*) which predominantly affects poor and vulnerable populations. It is estimated that there are more than 7,500 deaths per year worldwide from CD, and that approximately six million people are infected with *T*. *cruzi*, most of them in endemic areas of Latin American countries [1,2]. In the Americas, CD is the parasitic disease with the highest mortality burden and disability-adjusted life years (DALYs) [2]. In Brazil, the prevalence of CD is around 0.6%, which corresponds to more than 1.1 million infected individuals [3]. The country represents one of the main endemic areas for CD in the world [4]. Minas Gerais is a Brazilian state endemic for CD [5–7] and this state has the highest number of deaths from CD in Brazil [7,8]. About 30% of these deaths are registered in two of the 12 regions of the state: Norte de Minas and Vale do Jequitinhonha [8]. These regions also have the worst socioeconomic indicators in the state of Minas Gerais [9]. The SamiTrop Project is a multicenter cohort that monitors almost two thousand patients with CD residing in these two regions [6]. Preliminary results from this cohort showed that 74% of these patients were not followed up by a doctor or were followed up irregularly [10].

Although the annual incidence and prevalence rates of CD have fallen as a result of control measures and improvements in quality of life, care to patients with CD is still a challenge for health systems. Studies point out barriers to the diagnosis and treatment of CD in endemic [11,12] and non-endemic countries [13,14]. In Brazil, the recommendation is that individuals with CD be followed up longitudinally in public primary health care (PHC) services through periodic medical consultations and, when necessary, be referred to specialized health services [15]. The expansion of public PHC services in the country to remote and disadvantaged areas, through the Family Health Strategy (FHS), has facilitated the access of patients with CD to medical care, however this is in a context where care gaps remain, with insufficient public funding and inadequate distribution of services. Currently, the FHS is the main PHC model in the country. The FHS provides health care for the population of a defined territory, and the services are provided by a team that includes a family doctor [16].

Without a national policy for attention to CD patients in Brazil, family health teams assume the responsibility of caring for these patients without the support of an organized and financed care network [17]. In this context, the work of the family doctor is decisive for adequate care for these patients. Taking into account the need for studies on care to patients with CD in different settings, this study explored the challenges of family doctors to provide care to patients with CD in an endemic region in Brazil with high coverage of public PHC services. In addition, this study has generated important reflections on the adequate clinical management of CD in PHC.

## Methods

### Study design

This study used a qualitative research method and a focus group (FG) as a technique for data collection. The qualitative method allowed to explore and understand the challenges of family doctors to provide care to patients with CD. The production of the data occurred in the FG through interaction between the participants of a group interview [18]. It is noteworthy that this study occurred as nested in the SamiTrop cohort study project [6].

### Research environment

**Fig 1:**
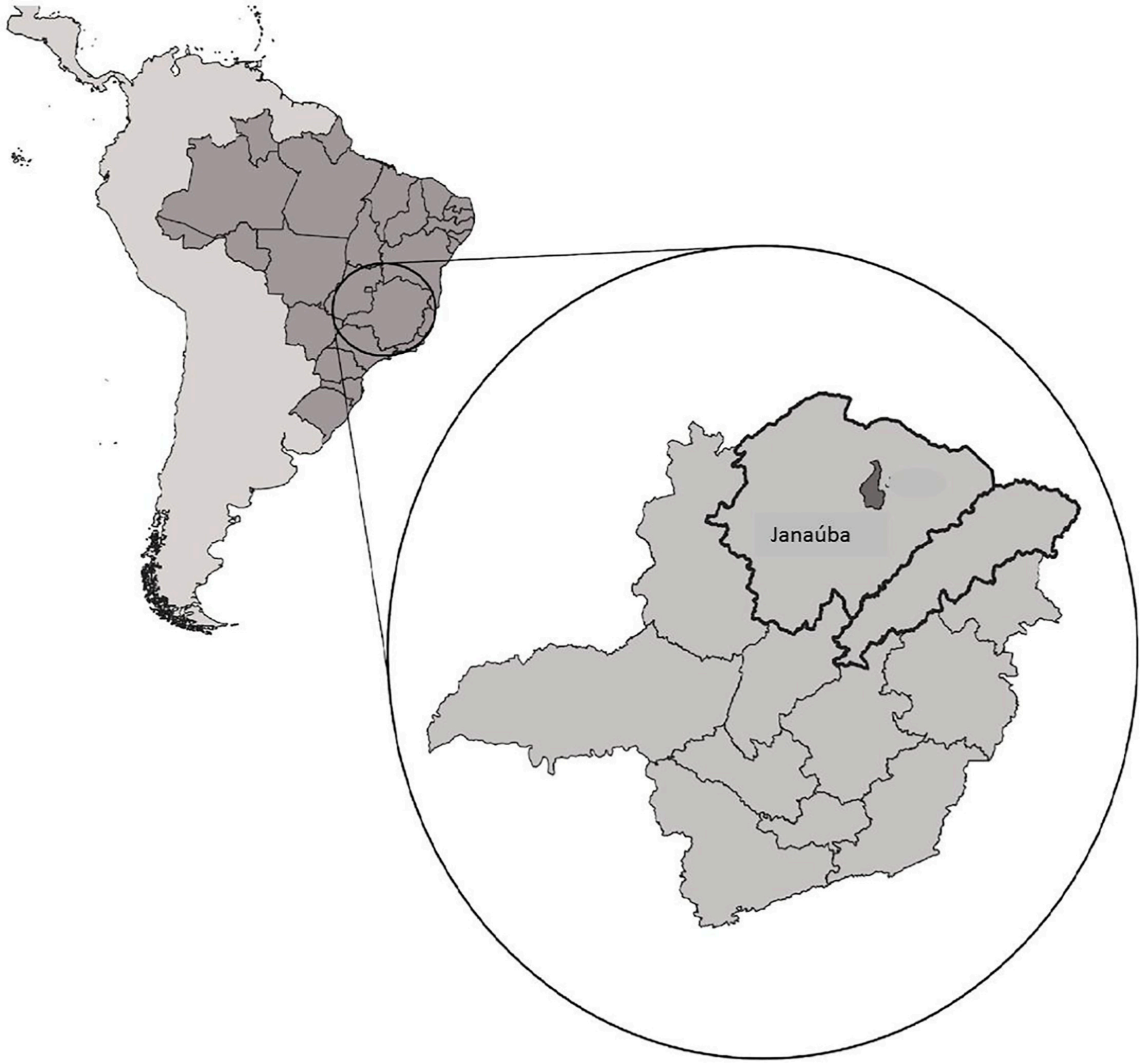
Location of the two regions of the state of Minas Gerais, Brazil, where the municipalities selected by the SaMiTrop cohort are located.

This study was carried out in the municipality of Janaúba, located in the state of Minas Gerais, Brazil. Janaúba is one of the municipalities participating in the SaMiTrop project (Center for Research on Biomarkers in Neglected Tropical Diseases in São Paulo/Minas Gerais). The SamiTrop project is a multicenter cohort that follows almost two thousand patients with CD in 21 municipalities located in two regions of the state of Minas Gerais (Fig 1). The cohort is funded by the National Institutes of Health (NIH) [6].

Although there is no knowledge of the prevalence of CD in the municipalities of these two regions, not even on the part of the municipal health secretariats, among the municipalities participating in the SamiTrop Project, Janaúba had the highest number of deaths from CD in the last ten years [8]. The municipality represents the scenario of small municipalities in regions endemic for CD in Brazil. Janaúba has a population of 66,803 inhabitants. Around 10% of the municipality's population is rural, the Human Development Index (HDI) is 0.696 (mean), and more than 93% of the population depends on the public health system [9]. The health system in Janaúba is organized into three levels of care (primary care, specialized outpatient care, and hospital care). Like many municipalities in the interior of Brazil, Janaúba has 100% coverage of PHC public services [19]. The municipality has 23 family health teams deployed in 13 health units. There are 10 health units in the urban area and three health units in rural areas. There are no specialized reference centers for CD in the regions of the state of Minas Gerais [20].

### Recruitment and participants

The sample of this study was qualitative and intentional. All 23 doctors from the family health teams in the municipality were invited to participate in the study. The invitation was made with the support of the PHC Municipal Coordinator. Fifteen doctors participated in the FG and composed the investigated sample.

### Data collection

Since only 15 doctors accepted to participate in the study, we opted to conduct only one FG. The size of the FG should allow for the effective participation of the participants and the appropriate discussion of the themes [18]. In the FG with 15 family doctors, it was possible to comply with these two recommendations.

The FG occurred in 2017 in the meeting room of the municipality's health department and lasted a little over two hours. It was conducted by four researchers. One researcher acted as moderator, two researchers acted as rapporteurs and one as an observer. The discussions in the FG were guided by the theme “medical care to patients with CD in PHC”. The results of a quantitative study previously carried out by researchers from the SamiTrop Project with PHC doctors from the same region [21] helped the moderator to guide the discussions. The FG was only closed when the doctors' speeches became repetitive and predictable, presenting nothing new in terms of content and arguments [18]. Sociodemographic variables (gender, age, time since graduation, university where they graduated, and postgraduate studies) were collected to characterize the participants. The meeting was recorded and all speeches were transcribed in full, constituting the analysis material.

### Data analysis

Data analysis was performed using the thematic content analysis technique. The steps were carried out as follows: pre-analysis of the content of the transcribed speeches, coding, treatment of results, inference, and interpretation.

Two researchers reviewed the transcribed speeches and codified the speeches based on the content and themes to generate categories of analysis. The categories of analysis generated were discussed with a third researcher, and subsequently grouped semantically into four categories. The statements of the FG participants who best illustrated the four categories were chosen to compose the results of this study. The results were interpreted from these categories and based on the scientific literature. The statements presented in the results were identified through the doctor's time of academic education. Complements were inserted to the speeches in order to clarify the context, when necessary.

### Ethical approval

This study was carried out in accordance with the current Brazilian legislation on research involving human beings [22]. It was approved by the Research Ethics Committee of the State University of Montes Claros (opinion No. 1,175,485). All participants in this study signed the Free and Informed Consent Form.

## Results

The FG with 15 family doctors was conducted. The average age of the participants was 33.4 years; 53.3% were female; 73.3% had less than 5 years of graduation. The average time the professionals graduated was 6.5 (± 8.06) years, with a minimum of one year and a maximum of 30 years. Of the doctors, 93.3% had graduated from universities in the state of Minas Gerais and only 33.3% had a medical residency or specialization: family health (3), hematology (1), and forensic medicine (1). The family doctors reported that care to patients with CD in PHC occurred only based on the demands of patients who sought medical consultation. They also reported that they did not know how many patients with CD there were in the municipality and that there were no records of these patients in health units.

Regarding the challenges to medical care for patients with CD in PHC, four themes emerged from the speeches of family doctors participating in the FG: unsatisfactory medical training for the care of patients with CD; uncertainties regarding antiparasitic treatment in the chronic phase of the disease; difficulty of patients' access to specialized care; and the trivialization of the disease by patients as a barrier to seeking care.

### Unsatisfactory medical training for care to patients with CD

In general, the family doctors reported that the university did not adequately prepare them for the clinical management of CD (academic education failure). They also reported that there was no offer of training for PHC doctors on CD. There was no disagreement between the doctors' reports according to the time of academic education.

“At the least my academic education was very flawed in relation to CD. At graduation, I studied the vector, protozoa, wattle and daub house and the repercussions. But like this, dealing with CD, the protocol, how often the patient has to be monitored, how often the tests are performed, when referring the patient, this was very flawed.” (Doctor graduated 3 years ago)

“Our training is flawed. We do not have a broad knowledge to follow these patients as they should be followed…” (Doctor graduated 1 year ago)

“During the time that the permanent education program for family doctors worked in the municipality, there was a lot of talk about leishmaniasis, tuberculosis, but CD was not talked about.” (Doctor graduated 7 years ago).

In addition, the family doctors pointed out that they needed to improve in relation to care to patients with CD in the chronic phase. However, there were reports that indicate the non-recognition of the doctor’s responsibility in the search for knowledge to improve care for patients with CD.

“We in primary care do have to improve our care for patients with CD in the chronic phase, how to deal with the complications of problems, with medications. I think that PHC needs improvement, however, I also agree with colleagues about the lack of a permanent education program on CD.” (Doctor graduated 3 years ago)

“I was researching the treatment of chronic CD to give an answer to the patient who asked about the treatment (…), but until today I have not researched…” (Doctor graduated 2 years ago)

### Uncertainties regarding antiparasitic treatment in the chronic phase of the disease

Family doctors have demonstrated uncertainties regarding antiparasitic drug treatment in the chronic phase of CD. Both recently trained doctors and doctors who have been trained for a long time pointed out that they were unaware of the recommendations for the treatment of chronic CD with the use of Benznidazole (BZN). All doctors participating in this study reported a lack of prior experience in prescribing BZN for the treatment of patients with chronic CD.

“In the acute phase, there is a chance that treatment with BZN will actually lead to a cure, when the disease is discovered early and the protocol is followed correctly. Now, in the chronic phase, my opinion is really one of uncertainty. I do not know if there would be any benefit after the clinical manifestations were installed. I also don't know if for the chronic patient, the treatment would increase survival. I can't say.” (Doctor graduated 3 years ago)

“I am aware that BZN treatment is for the acute phase, to try, as the colleague said, to reduce parasitemia. But I have no knowledge about the use and adverse reactions. I don't have a very well-formed concept about it.” (Doctor graduated 3 years ago)

“The guidance I had (…) was that treatment with BZN should be performed at any stage, chronic or acute, which at any stage would benefit. I don't know how it is today (…), I don't know exactly how it is.” (Doctor graduated 30 years ago)

“It is no longer CD. I don't know, sometimes I may be talking nonsense.” (Doctor graduated 20 years ago)

“Once, a young patient with a CD asked me if she would be advised to use BZN… I said that from what I know of the disease, there would be no indication, because her serology has been positive for many years.” (Doctor graduated 2 years ago)

### Difficulty in accessing specialized care

The family doctors expressed suffering and anguish for not being able to guarantee the access of patients with the cardiac form of CD to the cardiologist, and to some tests in the public health system, when necessary. There was no disagreement as to the recognition of the limitations in the care of these patients in PHC.

“We follow up, investigate, order tests if necessary, but is there a complication? Need to forward? Who will you forward to? So we stop at this point, because we have no one to refer to. There would be a private service, but we are referring to a poorer population, a population that has greater financial difficulties, especially when talking about the rural area, where the incidence of CD is greater.” (Doctor graduated 1 year ago)

“(…) we end up having difficulty referring to the cardiologist, because the vacancies are very limited and the patients end up being monitored in PHC.” (Doctor graduated 7 years ago)

“(…) I have a lot of difficulty monitoring patients with the cardiac form of the CD correctly, following the necessary protocols…” (Doctor graduated 3 years ago)

“The chronic chagasic patient with cardiac complications should be monitored by the cardiologist (…). Here in the city, this access is flawed, places for consultation with a specialist doctor and for exams are limited. This is a chronic problem.” (Doctor graduated 1 year ago)

“Patients with CD do not have follow-up with access to exams, to specialists. So this is flawed. We have no support, we do not have extensive knowledge to follow these patients as they should be followed.” (Doctor graduated 1 year ago)

### Trivialization of the disease by patients as a barrier to the search for care

The reports of family doctors pointed out that the patients they attended having CD was common, trivial, and typical of the environment where they lived. There was no disagreement regarding this perception among doctors.

“In rural areas, almost all people aged 30 or 40 have positive CD serology (…) People know whether or not they have the disease. But it seems that this information is not assimilated by them.” (Doctor graduated 20 years ago)

“(…) we ask, do you have CD? (the patient replies) I have. (…) So they know they have CD. But it seems that they do not relate the clinical manifestations to the diagnosis of the disease. So, the problem here is not the diagnosis, at some point this was done. The problem is what this diagnosis means to the patient!” (Doctor graduated 4 years ago)

According to family doctors, in general, patients with CD could not relate the clinical manifestations to the disease, even knowing the diagnosis. They also reported that these patients, even though they knew they had the disease, only go to the health service when they had some complication.

“Most cases already arrive with the diagnosis of the disease and have complications. They say: I have CD in my blood! So, even with intestinal or cardiac involvement, patients do not make this relationship.” (Doctor graduated 7 years ago)

## Discussion

The results of this study showed that family doctors in a municipality in an endemic region with high coverage of public PHC services face the following challenges in care for patients with CD: unsatisfactory medical training; uncertainties regarding antiparasitic treatment in the chronic phase of the disease; difficulty of patients' access to specialized care; and the trivialization of the disease by patients as a barrier to seeking care.

The family doctors participating in this study had a similar profile to PHC doctors in Brazil, being young professionals, recently graduated and with their first professional experience in PHC [23,24]. According to the report of these doctors, although they treat many patients with CD, the disease is a hidden problem for PHC. We observed that the lack of records of patients with CD in health units was one of the main factors that contributed to this. The lack of records on CD contributes to the invisibility of the disease in health systems. The records of patients with CD is the starting point not only for clinical follow-up, but also for planning health actions, and consequently, for allocating resources and assessing the impact of health care [25].

Regarding the unsatisfactory medical training for the care of patients with CD, family doctors considered that the lack of knowledge regarding the clinical management of CD is related to deficiencies in academic education and the lack of training on CD for PHC doctors. We emphasize that most of these doctors took the undergraduate course in higher education institutions in the state of Minas Gerais, an endemic region for CD [5–7]. This finding suggests that in the academic education of these doctors, epidemiological aspects of the region were not considered, as recommended by the Brazilian Curricular Guidelines [26]. As for the report of absence of training, there were no records of strategies adopted by the Brazilian Ministry of Health or by the State Department of Health of Minas Gerais for offering training on CD for PHC doctors [27,28].

Studies carried out in endemic and non-endemic countries [11,13,14,29–32] have also shown health professionals' lack of knowledge regarding CD. This lack of knowledge was identified as a barrier for access to adequate assistance in Colombia [11], Argentina [29] and the USA [14]. Family doctors in Madrid acknowledged having little knowledge about CD, but expressed a proactive attitude in the search for knowledge [13]. In general, the family doctors participating in our study, even working in an endemic region, did not recognize their own responsibility for seeking knowledge to improve care for patients with CD, or when they did, they did not seek knowledge. Perhaps the biggest flaw in the academic education of these doctors is the lack of encouragement to adopt an active stance in the search for knowledge. We emphasize that Brazilian doctors have had consensus that standardizes the strategies for diagnosis, treatment, prevention, and control of CD in the country since 2005 [4,33]. In 2018, the Clinical Protocol and Therapeutic Guidelines (CPTG) of CD in Brazil was published [15]. As much as the Brazilian public health system should contribute to the training of these professionals, it is up to the doctor to continually improve their knowledge and use the best of scientific progress for the benefit of the patient and society [34].

Regarding the uncertainties of family doctors for antiparasitic treatment in the chronic phase of CD, which led to the lack of timely treatment of patients in PHC, studies carried out in other municipalities in Brazil pointed out that the doctor’s lack of knowledge in relation to antiparasitic treatment of chronic CD was also the main reason for not prescribing BZN for patients who could benefit from treatment [21,35]. In Colombia and Argentina, the low level of knowledge of doctors was also considered as one of the barriers to antiparasitic treatment of CD [11, 29].

Antiparasitic treatment of chronic CD with BZN can slow the progression of the disease and prevent complications, however, this is associated with a high incidence of adverse events, especially in adults, and the benefits are uncertain for particular groups [15,36–38]. As the reduction in parasitic burden and the clinical, social, and economic benefits outweigh the negative aspects of antiparasitic treatment of CD in the chronic phase, PHC is the ideal scenario to guarantee access to early diagnosis and timely treatment of the disease for the population [38,39,40]. However, PHC doctors must know the recommendations for treatment, in addition to knowing how to identify and manage adverse events [15,25,38].

In order to increase the knowledge of PHC doctors regarding assistance to patients with CD, and to reduce uncertainties regarding antiparasitic treatment in chronic CD, the implementation of teaching on CD in the curricula of undergraduate courses in the health area should be strongly recommended, especially in endemic countries. In addition to offering specific training on CD, strategies such as the widespread dissemination of the CD CPTG, the creation of applications for easy access to the CPTG and the provision of decision support systems for clinical management of the disease should also be considered. Platforms for e-learning are also a valuable tools to expand access to medical education, as well as online access to guidelines, and medical updates [25].

Regarding the difficult access of the patient with CD to specialized care, this study showed that family doctors assumed the responsibility of caring for the patient with the cardiac form of CD without the support of a specialized service. This situation generated a feeling of helplessness in doctors and could have compromised patient care. In the region where the study was carried out, there is no reference center for CD and family doctors reported difficulties for patients to access the cardiologist. On the other hand, PHC doctors in that region have the offer of the tele-electrocardiogram service. This service makes it possible for doctors to recognize cardiac involvement early in chronic conditions by means of electrocardiogram (ECG) reports performed remotely [41]. The initial cardiac evaluation of the patient with CD can be performed in PHC, however, when there are changes in the ECG, evaluation by a cardiologist is necessary [25].

The difficulty in accessing specialized health services in Brazil is a chronic problem in the public health system that does not only affect patients with CD. In order to guarantee the access of the patient with CD to specialized care, in addition to the provision of services, it is necessary to promote the integration between PHC and specialized care [16]. A study carried out in São Paulo, Brazil, confirmed the feasibility of managing chronic CD in PHC, however this is in a context in which there were, in addition to trained doctors and the adoption of clinical guidelines, the existence of reference centers for specialized diagnostic and assistance support when necessary [42]. The creation of reference centers for PHC linked to CD was also recommended for non-endemic countries. These centers, in addition to providing diagnostic support and clinical consultations, could also provide health education and training activities for professionals [14]. To enable the access of patients with CD in remote areas to specialized care, strategies such as telemedicine and mobile clinics must be considered [25]. The implementation of care networks for people with CD may contribute to comprehensive care, especially in endemic regions.

Regarding the trivialization of the disease by patients as a barrier in the search for care, according to family doctors, as patients considered CD as something common, trivial, typical of the environment where they lived, the search for medical care occurred only when the patient presented some complication. Systematic review of qualitative studies on the socio-cultural aspects of CD also found that in endemic areas, the absence of symptoms and impact on the daily activities of CD patients contributed to the naturalization and normalization of CD, which influenced the search for care [43]. Studies with Bolivians with CD revealed that the disease was perceived as a common and even relatively harmless condition [44,45].

Family doctors participating in our study also reported that in general, patients with CD could not relate the clinical manifestations to the disease, even though they knew the diagnosis. In Argentina the lack of information about CD also influenced the demand for care [46]. A study carried out in Spain pointed out that the low understanding of patients with CD in relation to the disease was not exclusively related to the patients' level of education, but also to the difficulties surrounding doctor-patient communication [47]. In our study, we observed that family doctors' lack of knowledge about the clinical management of the disease and the lack of recognition of CD as a local problem, are factors that may have compromised doctor-patient communication and patients' understanding of the disease and its complications.

In addition to the doctors' awareness of the disease and its social consequences, it is necessary that the PHC services offer support for patients with CD to face the emotional and social challenges experienced by them. These patients must be monitored by a multidisciplinary team with the presence of a psychologist and social worker. The creation of associations of people with CD in communities and initiatives such as The Catalonian Expert Patient Program on Chagas Disease should be adopted to increase patients' knowledge about the disease, provide social and emotional support to patients, and promote self-care [25, 48].

### Strengths and limitations

This is the first qualitative study conducted in an endemic region with a high coverage of public PHC services in Brazil that explored the challenges of family doctors to provide care to patients with CD in PHC. Although the results of this study are not generalizable in other contexts, they provide important reflections. The realization of the FG in a single municipality was a limitation. However, the duration of the FG and the profile of the participants (many had experiences as family doctors in other municipalities in the region) contributed to the collection of consistent material with great analytical potential.

## Conclusions

Access to adequate medical care for CD patients, even in regions with high coverage of public PHC services, still represents an important challenge for health systems. The challenges identified in this study may contribute to the development of strategies to improve the clinical management of CD in PHC.

## Supporting information

S1 Appendix Complete and Portuguese speeches of family doctors. (DOC)

## References

[1] World Health Organization. Integrating neglected tropical diseases into global health and development: fourth WHO report on neglected tropical diseases. Geneva: World Health Organization; 2017 Available from: http://apps.who.int/iris/bitstream/10665/255011/1/9789241565448-eng.pdf.

[2] World Health Organization. Global Health Estimates 2016: Disease burden by Cause, Age, Sex, by Country and by Region, 2000–2016. Geneva: World Health Organization; 2018.

[3] World Health Organization. Chagas disease in Latin America: an epidemiological update based on 2010 estimates. Geneva: World Health Organization; 2015. 25671846

[4] Dias JCP, Ramos AN Jr, Gontijo ED, Luquetti A, Shikanai-Yasuda MA, Coura JR, et al 2 nd Brazilian Consensus on Chagas Disease, 2015. Rev Soc Bras Med Trop. 2016; 49(1):3–60. 10.1590/0037-8682-0505-2016 27982292

[5] Marcolino MS, Palhares DM, Ferreira LR, Ribeiro AL. Electrocardiogram and Chagas disease: a large population database of primary care patients. Glob Heart. 2015; 10(3):167–172. 10.1016/j.gheart.2015.07.001 26407512

[6] Cardoso CS, Sabino EC, Oliveira CD, de Oliveira LC, Ferreira AM, Cunha-Neto E, et al Longitudinal study of patients with chronic Chagas cardiomyopathy in Brazil (SaMi-Trop project): a cohort profile. BMJ Open. 2016; 10.1136/bmjopen-2016-011181 27147390 PMC4861110

[7] Ministério da Saúde, Secretaria de Vigilância em Saúde. Doença de Chagas: 14 de abril–Dia Mundial. Bol Epidemiol [Internet]. 2020 4 [cited 2020 May 10]; 51(n.esp.):1–43. Available from: http://www.saude.gov.br/boletins-epidemiologicos.

[8] Ministério da Saúde, Secretaria de Vigilância em Saúde, Coordenação-Geral de Informações e Análises Epidemiológicas [Internet]. Sistema de Informações sobre Mortalidade. [cited 2020 May 10]. Available from: http://tabnet.datasus.gov.br/cgi/deftohtm.exe?sim/cnv/obt10uf.def.

[9] Instituto Brasileiro de Geografia e Estatistica-IBGE [Internet]. Censo demográfico 2010. Características da população e dos domicílios: resultados do universo. [cited 2020 May 25]. Available from: https://censo2010.ibge.gov.br/.

[10] Damasceno RF, Ferreira AM, Vieira TM, Campos MCOA, Leite SF, Moreira JM, et al. Uso dos serviços de saúde por pessoas com doença de Chagas: Projeto SaMi-Trop. Proceedings of the MEDTROP-Parasito 2019–55° Congresso da Sociedade Brasileira de Medicina Tropical; 2019 July 28–31; Belo Horizonte, Brazil.

[11] Marchiol A, Forsyth C, Bernal O, Valencia C, Cucunubá Z, Pachón E, et al Increasing access to comprehensive care for Chagas disease: development of a patient-centered model in Colombia. Rev Panam Salud Publica. 2017;41:153 10.26633/RPSP.2017.153 31384272 PMC6645187

[12] Alonso-Padilla J, Cortés-Serra N, Pinazo MJ, Bottazzi ME, Abril M, Barreira F, et al Strategies to enhance access to diagnosis and treatment for Chagas disease patients in Latin America. Expert Review of Anti-infective Therapy. 2019;17:145–157. 10.1080/14787210.2019.1577731 30712412

[13] Iglesias-Rus L, Romay-Barja M, Boquete T, Benito A, Blasco-Hernández T. The role of the first level of health care in the approach to Chagas disease in a non-endemic country. PLoS Negl Trop Dis. 2019;13(12):e0007937 10.1371/journal.pntd.0007937 31841503 PMC6913928

[14] Forsyth C, Meymandi S, Moss I, Cone J, Cohen R, Batista C. Proposed multidimensional framework for understanding Chagas disease healthcare barriers in the United States. PLoS Negl Trop Dis. 2019;13(9):e0007447 10.1371/journal.pntd.0007447 31557155 PMC6762052

[15] Ministério da Saúde (Brasil). Portaria n° 57, de 30 de outubro de 2018. Torna pública a decisão de aprovar o Protocolo Clínico e Diretrizes Terapêuticas da doença de Chagas, no âmbito do Sistema Único de Saúde—SUS. Diário Oficial da União 31 out 2018; Seção 1, p. 41.

[16] Castro MC, Massuda A, Almeida G, Menezes-Filho NA, Andrade MV, de Souza Noronha KVM, et al Brazil's unified health system: the first 30 years and prospects for the future. Lancet. 2019;394(10195):345–356. 10.1016/S0140-6736(19)31243-7 31303318

[17] Ministério da Saúde (Brasil). Portaria de Consolidação n° 2, de 28 de setembro de 2017. Consolidação das normas sobre as políticas nacionais de saúde do Sistema Único de Saúde. Diário Oficial da União.

[18] Krueger RA, Casey MA. Focus groups: A practical guide for applied researchers 4rd ed. Thousand Oaks: CA; 2008.

[19] Ministério da Saúde [Internet]. Informação e Gestão da Atenção Básica. [cited 2020 May 15]. Available from: https://egestorab.saude.gov.br/paginas/acessoPublico/relatorios/relHistoricoCoberturaAB.xhtml.

[20] Ministério da Saúde [Internet]. Cadastro Nacional de Estabelecimentos de Saúde [cited 2020 May 15]. Available from: http://cnes.datasus.gov.br/.

[21] Ferreira AM, Sabino EC, Moreira HF, Cardoso CS, Oliveira CL, Ribeiro ALP et al Rev. APS. 2018;21(3):345–354.

[22] Ministério da Saúde (Brasil). Resolução n. 466, de 12 de dezembro de 2012. Aprova diretrizes e normas regulamentadoras de pesquisas envolvendo seres humanos. Diário Oficial da União 13 jun. 2013; Seção 1, p. 59.

[23] Damasceno RF, Caldeira AP. Factors associated with the non-use of telehealth consultancy by physicians of the Family Health Strategy. Ciênc. saúde coletiva. 2019;24(8):3089–3098. 10.1590/1413-81232018248.28752017 31389555

[24] Conselho Federal de Medicina, Conselho Regional de Medicina do Estado de São Paulo, Departamento de Medicina Preventiva da Faculdade de Medicina da USP. Demografia Médica no Brasil 2018. São Paulo (SP); 2018.

[25] Echeverría LE, Marcus R, Novick G, Sosa-Estani S, Ralston K, Zaidel EJ, et al WHF IASC Roadmap on Chagas Disease. Global heart. 2020;15(1):26 10.5334/gh.484 32489799 PMC7218776

[26] Ministério da Educação (Brasil). Resolução n° 3, de 20 de junho de 2014. Institui Diretrizes Curriculares Nacionais do Curso de Graduação em Medicina e dá outras providências. Diário Oficial da União 23 jun. 2014; Seção 1, p. 8–11.

[27] Ministério da Saúde (BR) [Internet]. Doença de Chagas: o que é, causas, sintomas, tratamento e prevenção [cited 2020 May 20]. Available from: http://saude.gov.br/saude-de-a-z/doenca-de-chagas.

[28] saude.mg.gov.br [Internet]. Secretaria de Estado de Saúde de Minas Gerais (BR). [cited 2020 May 20]. Available from: https://www.saude.mg.gov.br/.

[29] Klein K, Burrone MS, Alonso JP, Rey Ares L, García Martí S, Lavenia A, et al Estrategia para mejorar el acceso al tratamiento etiológico para la enfermedad de Chagas en el primer nivel de atención en Argentina. Rev Panam Salud Publica. 2017; 10.26633/RPSP.2017.20 28591327 PMC6660878

[30] Berger BA, Bartlett AH, Jiménez-Hernández R, Trinidad Vázquez E, Galindo-Sevilla N. Physician Knowledge, Attitudes, and Practices Related to Chagas Disease in Tabasco, Mexico. Am J Trop Med Hyg. 2018;98(6):1743–1747. 10.4269/ajtmh.17-0495 29692299 PMC6086191

[31] Amstutz-Szalay S. Physician Knowledge of Chagas Disease in Hispanic Immigrants Living in Appalachian Ohio. J Racial Ethn Heal Disparities. 2017;4:523–528. 10.1007/s40615-016-0254-8 27324820

[32] Montgomery SP, Parise ME, Dotson EM, Bialek SR. What Do We Know About Chagas Disease in the United States? Am J Trop Med Hyg. 2016;95:1225–1227. 10.4269/ajtmh.16-0213 27402515 PMC5154432

[33] Ministério da Saúde, Secretaria de Vigilância em Saúde. Consenso Brasileiro em Doença de Chagas. Rev Soc Bras Med Trop. 2005;3:1–29. 16416933

[34] Conselho Federal de Medicina (Brasil). Resolução n° 2.217/2018, de 27 de setembro de 2018. Aprova o Código de Ética Médica. Diário Oficial da União 01 nov 2018; Seção I, p.179.

[35] Colosio RC, Falavigna-Guilherme AL, Gomes ML, Marques DSO, Lala ERP, Araújo SM. Conhecimentos e atitudes sobre a doença de Chagas entre profissionais de saúde–Paraná, Brasil. Ciência, Cuidado E Saúde. 2008;6:355–363.

[36] Cardoso C, Ribeiro A, Oliveira C, Oliveira L, Ferreira A, Bierrenbach A, et al Beneficial effects of benznidazole in CD: NIH SaMi-Trop cohort study. PLoS Negl Trop Dis. 2018;12(11). 10.1371/journal.pntd.0006814 30383777 PMC6211620

[37] Camara EJN, Mendonca VRR, Souza LCL, Carvalho JS, Lessa AS, Gatto R, et al Elevated IL-17 levels and echocardiographic signs of preserved myocardial function in benznidazole-treated individuals with chronic Chagas' disease. Int J Infect Dis. 2019;79:123–130. 10.1016/j.ijid.2018.11.369 30528394

[38] Pan American Health Organization (PAHO). Guidelines for the diagnosis and treatment of Chagas disease. Washington, DC; 2019.

[39] Requena-Méndez A, Bussion S, Aldasoro E, Jackson Y, Angheben A, Moore D, et al Cost-effectiveness of Chagas disease screening in Latin American migrants at primary health-care centres in Europe: a Markov model analysis. Lancet Glob Health. 2017;5(4):e439–e447. 10.1016/S2214-109X(17)30073-6 28256340

[40] Bartsch SM, Avelis CM, Asti L, Hertenstein DL, Ndeffo-Mbah M, Galvani A, et al The economic value of identifying and treating Chagas disease patients earlier and the impact on Trypanosoma cruzi transmission. PLoS Negl Trop Dis. 2018; 12(11):e0006809 10.1371/journal.pntd.0006809 30395603 PMC6237415

[41] Marcolino MS, Alkmim MB, Santos TADQ, Ribeiro AL. The telehealth network of Minas Gerais: a large-scale Brazilian public telehealth service improving access to specialised health care. Policy in Focus. 2016;13(1):59–61.

[42] Shikanai Yasuda MA, Sátolo CG, Carvalho NB, Atala MM, Ferrufino RQ, Leite RM, et al Interdisciplinary approach at the primary healthcare level for Bolivian immigrants with Chagas disease in the city of São Paulo. PLoS Negl Trop Dis. 2017; 11(3):e0005466 10.1371/journal.pntd.0005466 28333923 PMC5380346

[43] Ventura-Garcia L, Roura M, Pell C, Posada E, Gascón J, Aldasoro E, et al Socio-Cultural Aspects of CD: A Systematic Review of Qualitative Research. PLoS Negl Trop Dis. 2013; 7(9):e2410 10.1371/journal.pntd.0002410 24069473 PMC3772024

[44] Blasco-Hernández T, García-San Miguel L, Navaza B, Navarro M, Benito A. Conhecimento e experiências da doença de Chagas em mulheres bolivianas que vivem na Espanha: um estudo qualitativo. Ação de Saúde Glob. 2016;9:30201 10.3402/gha.v9.30201 26976265 PMC4789531

[45] Forsyth CJ. "I Cannot Be Worried": Living with Chagas Disease in Tropical Bolivia. PLoS Negl Trop Dis. 2017;11(1):e0005251 10.1371/journal.pntd.0005251 28099488 PMC5242422

[46] Llovet I, Dinardi G, Canevari C, Torabi N. Atenção à saúde em busca de comportamentos de pessoas com doença de Chagas aguda na zona rural da Argentina: uma visão qualitativa. J Trop Med. 2016; 10.1155/2016/4561951 27829843 PMC5088329

[47] Muñoz-Vilches MJ, Salas-Coronas J, Gutiérrez-Izquierdo MI, Metz D, Salvador-Sánchez J, Giménez-Sánchez F. Conocimiento de la Enfermedad de Chagas por parte de los profesionales sanitarios de tres hospitales en la provincia de Almería. Rev Esp Salud Publica. 2013;87(3):267–75. 10.4321/S1135-57272013000300006 23892678

[48] Claveria Guiu I, Caro Mendivelso J, Ouaarab Essadek H, González Mestre MA, Albajar-Viñas P, Gómez IPJ. The Catalonian Expert Patient Programme for Chagas Disease: An Approach to Comprehensive Care Involving Affected Individuals. J Immigr Minor Health. 2017;19(1):80–90. 10.1007/s10903-016-0345-y 26895150

