# Supplementary material for: Challenges in the care of patients with Chagas disease in the Brazilian public health system: A qualitative study with primary health care doctors
Source: PLoS Negl Trop Dis. 2020 Nov 9;14(11):e0008782. doi: 10.1371/journal.pntd.0008782 (PMC7676681; doi:10.1371/journal.pntd.0008782)
Supplement: S1 Appendix — (DOC) [file pntd.0008782.s001.doc]

**Challenges in the care of patients with Chagas disease in the Brazilian public health system: a qualitative study with primary health care doctors**

**Complete and Portuguese speeches of family doctors presented in the study**

| **Unsatisfactory medical training for care to patients with CD** |
| --- |
| “Pelo menos a minha formação foi muito falha em relação a doença de Chagas. Na graduação, eu estudei sobre o vetor, protozoário, casa de pau a pique e as repercussões. Mas assim, lidar mesmo com a doença de Chagas, protocolo, de quanto em quanto tempo tem que ser feito o acompanhamento, qual a periodicidade dos exames, quando referenciar, isso foi muito falho.” (Médico formado há 03 anos)  “A nossa formação é falha. A gente não tem um conhecimento amplo para acompanhar esses pacientes como eles deveriam ser acompanhados na atenção primária.” (Médico formado há 01 ano)  “Durante o tempo em que o programa de educação permanente para médicos de família funcionou no município, foi falado muito sobre leishmaniose, tuberculose, mas sobre doença de Chagas não foi falado.” (Médico formado há 07 anos).  “Nós da atenção primária, temos sim que nos aprimorar na atenção aos paciente crônicos, como lidar até com as complicações dos problemas, quanto as medicações. Eu acho que precisa de aprimoramento da atenção primária, no entanto, eu também concordo com os colegas quanto à falta de um programa de educação permanente sobre chagas.” (Médico formado há 03 anos)  “Eu fiquei de pesquisar sobre o tratamento da doença de Chagas crônica para dar uma resposta ao paciente que perguntou sobre o tratamento. E isso não tem muito tempo, mas até hoje eu não pesquisei. Nessa correria do dia-a-dia, dou a mão a palmátoria, porque realmente eu não fui pesquisar. Eu fiquei de dar a resposta e até hoje nada.” (Médico formado há 02 anos) |
| **Uncertainties regarding antiparasitic treatment in the chronic phase of the disease** |
| “Na fase aguda tem uma chance do tratamento com benzonidazol realmente levar a cura, quando a doença for descoberta no início e o protocolo for seguido direito. Agora, na fase crônica, minha opinião é realmente de incerteza. Eu não sei se teria algum benefício depois de já instaladas as manifestações clínicas. Não sei também se para o paciente crônico, o tratamento aumentaria a sobrevida. Eu não sei dizer.” (Médico formado há 03 anos)  “Eu tenho conhecimento é que o tratamento com benzonidazol é para fase aguda, para tentar, como o colega falou, diminuir a parasitemia. Mas, eu não tenho conhecimento sobre o uso e reações adversas. Eu não tenho um conceito muito bem formado sobre isso.” (Médico formado há 03 anos)  “A orientação que eu tive lá em Belo Horizonte era que o tratamento com benzonidazol fosse feito em qualquer fase, crônica ou aguda, que em qualquer fase teria benefício. Eu não sei hoje como está tratando a doença nessa fase, não sei exatamente como está”. (Médico formado há 30 anos)  “Não se trata mais doença de Chagas. Não sei, às vezes posso estar falando bobagem.” (Médico formado há 20 anos)  “Uma vez uma paciente jovem com doença de Chagas me questionou se não teria indicação dela usar o benzonidazol. Eu falei que pelo que eu conheço da doença, não haveria indicação, porque a sorologia dela já era positiva há muitos anos.” (Médico formado há 02 anos) |
| **Difficulty in accessing specialized care** |
| “A gente acompanha, investiga, pede exames se necessário, mas a partir do momento, mas tem complicação? Precisa encaminhar? Vai encaminhar para quem? Então, pára neste ponto, porque não tem para quem encaminhar. Teria o serviço particular, mas a gente está se referindo a uma população mais carente, uma população que tem uma dificuldade financeira maior, principalmente quando se fala da zona rural, onde a incidência da doença de Chagas é maior.” (Médico formado há 01 ano)  “E aí a gente acaba tendo dificuldade de encaminhar pro cardiologista, porque as vagas estão bem limitadas e os pacientes acabam sendo realmente acompanhados na atenção primária. E aí tem os casos que volta e meia tem que internar, ir para o CTI.” (Médico formado há 07 anos)  “Bom, assim, eu tento colocar no início essa questão do cardiologista. Eu tenho muita dificuldade em saber fazer o acompanhamento do paciente já com a forma cardíaca de forma bem certinha, seguindo os protocolos que são necessários pro paciente.” (Médico formado há 03 anos)  “O paciente crônico com complicação cardíaca deveria ter um acompanhamento com o cardiologista e a gente daria um apoio contínuo “né”, na atenção primária, mas eu acho que deveria ter um acompanhamento com o cardiologista. Aqui na cidade esse acesso é falho, as vagas para consulta com médico especialista e para realização de exames são limitadas. Isso é um problema crônico.” (Médico formado há 01 ano)  “Os pacientes com doença de Chagas não têm um acompanhamento com acesso à exames, à especialistas. Então isso é falho. A gente não tem respaldo, a gente não tem conhecimento amplo para acompanhar esses pacientes como eles deveriam ser acompanhados.” (Médico formado há 01 ano) |
| **Trivialization of the disease by patients as a barrier to the search for care** |
| “Na zona rural, quase a totalidade das pessoas de faixas etárias a partir de 30 ou 40 anos tem sorologia positiva para doença de Chagas e a cardiopatia eu acho que, quando chega assim, a gente pede tudo. As pessoas sabem se tem ou não tem a doença. Mas parece que essa informação não é assimilada por elas.” (Médico formado há 20 anos)  “E muitos já tem o diagnóstico, muitos já tem, mas daí a gente pergunta, você tem doença de Chagas? Tenho. Mas parece que essa informação, ela não faz um sentido. Então eles sabem que têm doença de Chagas. Mas parece que eles não relacionam as manifestações clínicas com o diagnóstico da doença. Então, o problema aqui não é o diagnóstico, em algum momento isso foi feito. O problema é o que esse diagnóstico significa para o paciente!” (Médico formado há 04 anos)  “A maioria dos casos já chegam com o diagnóstico da doença e apresentam complicações. Eles falam assim: eu tenho chagas no sangue! Então, mesmo com comprometimento intestinal ou cardíaco, os pacientes não fazem essa relação”. (Médico formado há 07 anos) |
